# Selections and Abstracts

**Published:** 1903-05

**Authors:** 


### SELECTIONS AND ABSTRACTS.

### Finsen and His Light.

In the February number of McClure's magazine there is a series of appreciative articles upon the work of the Danish physician, Niels Finsen, who introduced phototherapy, in its modern form, to the medical world. A fellow countryman, Mr. Jacob A. Riis, of New York, writes of Dr. Finsen as a man. Mr. Riis first met Finsen when he was ill in the Commune Hospital at Copenhagen in 1899. He had known his father, who was a bishop in his own native town, and was therefore better able to appreciate the sterling qualities of the man. "When the fever had left me," said Mr. Riis, "I would sit in his little office down in the corner of the hospital grounds by the lake and watch the patients who had come in pain and gloom, go away, carrying in their faces the sunshine that had given them back their life and I came to look with a kind of reverential awe upon this patient, silent man whose every thought was for his suffering fellows while he calmly counted the hours to his own release from racking pain."

Cleveland Moffett writes the story of Finsen's achievement. He says: "The story of Finsen's achievement is another instance of success growing out of apparent failure and strength out of weakness. For after studying medicine for eight years at the Copenhagen University, he took his doctor's degree in 1890 (at the age of thirty) only to find himself so stricken in body, with heart, liver and digestive organs all affected, that it was out of the question for him ever to practice his profession. So he turned to the work that offered, and for three years filled the modest post of preceptor in anatomy at the university, his health continuing as bad as possible. Thus in 1893 the Finsen whose name to-day is celebrated through all the scientific world was a poor and obscure instructor in a little Danish city.

"During these three years, however, Finsen did more than teach anatomy; his spare time, his thoughts, and any strength he had after the day's work were occupied with observations and experiments destined soon to rob smallpox of its ugliest terror, the scarring of the face. Not that he started with any such aim or had smallpox particularly in mind at first; he started with light and a study of its physiological action. Can light do any *good* to the body, can light do any *harm* to the body? . . .

"It was a simple enough line of reasoning which led Finsen to his first discovery. He found if a number of earthworms are placed in an oblong box covered half with red glass and half with blue glass, they will always crawl away from the blue light and seek shelter in the red light. In blue light they are restless and ill at ease; in red light they lie still, perfectly content.

"Finsen took note also of a curious experiment with the chameleon, which consists in placing this little animal so that half of its body is under blue glass and the other half under red glass, the result being that one half of the chameleon turns almost black under the blue light, while the other half remains almost white under the red light. Which means, explained Finsen, that the chameleon uses its movable pigment cells to protect itself against the disagreeable effects of blue light."

The conclusion of Finsen, as a result of these experiments, was that only the violet or actinic rays have any decided physiological effect upon animal life. At this point in his researches he came across a pamphlet published in 1832 by Dr. Picton, of New Orleans, in which it is noted that some soldiers who suffered from smallpox, while confined in a dungeon, escaped without suppuration or scarring. It at once occurred to him that this immunity was due to the exclusion of the actinic rays of light. As a practical conclusion he suggested that smallpox patients be treated in rooms in which all but the red rays of light are excluded. This method was soon tried in Norway and Sweden and found very successful. With so much accomplished he continued his experiments in another direction.

"It was well known at this time that ordinary sunlight will destroy bacteria if these are long enough exposed to its action. Finsen now proceeded to show that this bactericidal action of light is almost entirely limited to the blue, the violet, and the ultra-violet rays (the green, yellow, and red being practically useless), and that this action is greatly intensified by focusing the light through lenses. Thus Finsen found that while unfocused light from a July sun in Copenhagen would kill plate cultures of the *bacillus prodigiosus* in an hour and a half, the same light concentrated through lenses, with the useless rays filtered out, would kill similar cultures in two or three seconds, and the same was true of other bacteria—they were almost instantly destroyed if exposed to concentrated actinic rays.

"Now, evidently you can cure any bacterial disease if you can destroy the bacteria that cause it, so the essential thing to know next was how far into the body these chemical rays could be made to penetrate for this business of bacteria-killing. If they could be sent through and through the body (as some credulous newspapers have imagined), then all diseases of bacterial origin, tuberculosis and the rest, must certainly be cured, but it was soon found that any such considerable penetration is impossible with the present resources of science. The depth of the radial action into the tissues is very shallow—a few millimeters at the most. It is true that the actinic rays will penetrate farther when concentrated by lenses, but not far enough to make them available against any but superficial diseases."

Dr. Finsen also found that electric light was more efficient than sunlight because richer in ultra-violet rays and that a part from which the blood has been pressed is more easily influenced, because the red color of the blood hinders the passage of the violet rays

"Gradually a girdle of limitations was established about the new field of investigation. For instance, there is a variety of baldness due to bacteria which, it was reasonable to think, might be cured by the chemical rays. And there is a form of superficial cancer due to bacteria which also fitted the conditions. And there are various diseases (some due to bacteria and some not) which seemed to call the experimenter with his healing electric lamp. What would the chemical rays do for measles or acne or lupus? These were questions that could only be answered after months of tests.

"Finsen began with lupus, a dreadful, disfiguring disease, usually of the face, that comes when the bacteria of tuberculosis attack the surfaces of the body instead of the lungs or deeper parts. There was no cure for lupus, and thousands of sufferers over the world (there were some 1,500 in Denmark alone) were condemned without hope to endure its slow ravages. Surgeons might cut away the affected parts, but some of the bacteria were almost sure to remain, so that the knife gave only temporary relief.

"Finsen's first patient was an engineer of Copenhagen, Neils Morgensen, who for eight years since the lupus declared itself had vainly tried whatever science could suggest for his relief. No less than twenty-five times, he told me, his face had been operated on, the right side being cut, scraped, burned with acids, seared with hot irons, and all to no avail. In the fall of 1895 the phototherapeutic treatment on Morgensen was begun. At first everything was very crude; a hand lense was used to concentrate the rays from an ordinary arc lamp, the red and ultra red being filtered out through blue water. For an hour or two hours, every day, this concentrated blue light was directed against the afflicted right cheek, Finsen himself holding the lens, aided by a medical student.

"The result came up to the fullest expectations. After the first treatment there was no more spread of the disease, but a steady closing in of the lupus patches and a lessening of the angry redness as healthy tissue formed. Within six months Niels Morgensen was free from his disease.

"Needless to say Finsen has made many advances in the use of the light. He soon discovered, for instance, that the ultra-violet or invisible rays at the blue end of the spectrum are much more efficacious in killing bacteria, say ten times more so, than are the visible violet rays, and this fact led him to abolish the blue-water filter which prevents the ultra-violet rays from passing, and to use instead clear water, which sufficiently absorbs the red and ultra-red rays that would otherwise burn the skin. He also substituted a lens of rock crystal for the one of ordinary glass used at first, since he found that rock crystal allows the ultra-violet rays to pass freely, while ordinary glass almost stops them. And he gradually increased the power of his electric lamp from twenty-five amperes up to fifty, to seventy, to eighty amperes, as in the lamps used now.

Of course the more powerful the arc light is the more abundant is the supply of actinic rays and the greater their penetration. And the only reason why Finsen has stopped at lamps of eighty amperes (that is about three times the intensity of ordinary street arc lamps) is because above that point it is impossible, as yet, to cool down the light so that a patient can bear it."

Mr. Moffett gives an interesting sketch of the Finsen Institute at Copenhagen and other writers in the same journal describe the use of the "light" in England and America. The following is said regarding its use and limitations:

"Already the Finsen lamps have been used with success for cancer in its small surface form (*Epithelioma cutaneum*), the records of twenty-two such cases showing ten cures, four still under treatment, and eight where the treatment was discontinued. Also, obstinate cases of acne have been cured, as well as the kind of bacterial baldness (*Alopecia areata*) mentioned above. Excellent reresults have been obtained in erysipelas and minor eruptions, and there is opening a wide and promising field of investigation as to the benefits of electric-light baths and sun-baths in various nervous diseases and in insanity. At the Finsen Institute there is a large room where naked patients walk around for a prescribed length of time under a powerful electric light. And the roof is built flat, with rows of little dressing-houses for sun-bath patients. Of precise results here, however, it is still too soon to speak—Finsen's attitude toward possibilities of the future being to say nothing until he is sure. But the work of phototherapy is marching on in many laboratories."

### MANAGEMENT OF NORMAL CASES OF LABOR.*

The questions involved in the management of a woman in labor will confront every practitioner of medicine, and his ability will be put to proof before his medical career has extended very far, and to a beginner there is much that is trying and embarrassing, especially when his every movement and action is watched and criticized (and I must say sometimes justly) by the old mother midwife.

*Read by J. J. Adams, M.D., before the Muldraugh Hill Medical Society, December 11, 1902.

It is well to recollect also that nature alone usually terminates labor with safety to both mother and child, but at the same time it should not be forgotten that at any moment dangerous complications may arise which should be immediately recognized and promptly dealt with; on this account it is very necessary for the obstetrician to have a thorough knowledge of all dangerous complications.

Having been called to a case of labor, the first question is, what shall I take with me? A fairly well-equipped obstetrician should have in his obstetric bag scissors, needles, suture material, needle-holder, hemostats, one box sterilized gauze, package of borated cotton, a bottle of carbolized vaseline, a bottle of aseptic silk ligature for cord, hypodermic syringe, with the customary pellets, a bottle of fluid extract ergot, an obstetric forceps, a box of hermetically sealed tubes of aseptic ergot for hypodermic use, chloroform, fountain syringe, box of talcum powder, bottle of ethereal antiseptic soap, and a nail-brush. Before making an examination the physician should always thoroughly wash his hands with soap and water, and then with an antiseptic solution, and before introducing the examining fingers should lubricate them with carbolized vaseline.

The diagnosis of labor is the first question that confronts the physician when first called to visit his patient, and some very unfortunate mistakes have been made in diagnosis.

Dr. Barton Cook Hirst, of Philadelphia, Pa., cites a case. One of his students, having been called hurriedly to see a patient, spent some fifteen minutes sterilizing his hands, and made a prolonged vaginal examination, much to the patient's surprise, as she had sent for him on account of rheumatism.

An instance occurred in our county in which one of our best physicians made a diagnosis of ovarian tumor, and advised an operation, but after consultation decided to await further developments, and after a short time I delivered the woman of twins.

The signs of labor are pains in the sacral region or abdomen, recurring at intervals of from five minutes to a half-hour, which come on suddenly and last for about a minute; sometimes we have a little blood-tinged mucus escaping from the vagina, but the most reliable sign is the dilatation of the os, and when the pain comes on you feel with the examining finger the pressure downward, and membranes and os made tense.

Having made a diagnosis of beginning labor, the presentation and position of the child must be made out by vaginal touch, auscultation, and palpation. We make the diagnosis of presentation by dividing the uterus into four equal parts by an imaginary line corresponding to the median line and a transverse line which passes through the middle of the uterus, and may or may not pass through the umbilicus, as its position varies in different individuals.

In vertex presentations the maximum intensity of the fetal heart-sounds are heard below the transverse line and to the right or left of the perpendicular line.

Face presentations: On the transverse line and to the right or left of the perpendicular line.

Breech presentations: Above the transverse line and to the right or left of the perpendicular line.

Shoulder presentations: On the perpendicular line midway between its point of intersection of transverse line and the pubes.

After assuring yourself that all is well, inquire into the condition of the patient's kidneys and bowels, and if the bowels have not acted, relieve them with an anema of from a pint to a quart of warm soapsuds. If this is not attended to labor may be delayed, and the danger of a tear in a greatly distended vagina is augmented. During the first stage of labor permit the patient to get up and walk around, making examinations as often as the necessities or indications of each case demand.

Before the woman is put to bed to stay, the mattress should be protected by an oilcloth or mackintosh, and over this a folded quilt or sheet, and over the bed-sheet I have placed another sheet of some clean cotton. The Kelly pad may be used with advantage. As labor advances, and the first stage is about to pass into the second, that is, after the os is completely dilated, always place a cloth or sponge next to the patient to catch the escape of the liquor amnii. In a primipara I rarely rupture the membranes, as they dilate the outlet gradually, and in that way help to avoid rupture; but in a multipara I usually rupture the bag of waters at the end of the first stage of labor by puncturing them between pains.

In the first stage of labor, when the pains are very painful—cutting pains—and doing very little good, to save the worry to my patient I sometimes give a little chloral or morphine to ease them. During the second stage of labor I keep my patient in bed, and when the head has reached the floor of the pelvis the patient may sit up in bed during the pains, with her feet against the footboard. I have her pull upon a sheet, or something attached to the foot of the bed, or if she prefers I let some one hold her hands. If the cervix be directed backward, and to one side, hook the fingers into it in the intervals of a pain, and draw it toward the center of the parturient canal.

Occasionally the anterior lip of the cervix becomes impacted between the head and the pubes. To overcome this, press up the anterior lip in the intervals of pains, and hold it above the head when the pain comes on. During labor, if the pains are short, and not of sufficient force, I sometimes give ten grains of quinine, and have the abdomen well rubbed with camphor, and if the abdomen is very pendulous, I fasten a band tightly around the upper part of it. During the latter part of the second stage of labor most authorities have their patients lie on their side, the knees drawn up, and a folded pillow placed between them.

It is a fact that in this position naturally there is less danger of rupturing the perineum, but I usually let my patient lie on her back, her legs and thighs semiflexed, and her knees well apart, and when the head has put the perineum well on the stretch I introduce the index and middle fingers of my examining hand into the rectum, and pull the perineum forward toward the symphysis, the thumb at the same time making pressure on the head so as to retard its progress. When the head is about to pass out I have my patient open her mouth, and breathe fast. If the uterine contractions are too severe, they may be controlled by giving an anesthetic (some use ether, but I prefer chloroform).

I always put off the use of an anesthetic in labor to as near the end of the second stage as I can. I never push it to complete anesthesia, only using it to modify the severity of the pains, as it may prolong labor and superinduce post-partum hemorrhage. The head may be delivered in the interval of pain by drawing the chin downward, lifting the perineum forward, thus preventing rupture. I do not believe a physician is ever justified in making an incision into the perineum to save a tear. As soon as the head is born make a sweep of the forefinger between the child's neck and maternal symphysis to see if the cord is around its neck. If so, make gentle traction, and draw the loop large enough to slip it over the child's head; after this the head of the child should be held in one hand, while the other, placed upon the abdomen, follows the uterus down as it descends, and forces out the body.

During the delivery of the shoulders the expulsion should usually be left to uterine contraction. The most common cause of delay at this stage is the arrest of the anterior shoulder under the symphysis; to liberate this make traction directly downward. It may also become necessary to assist the expulsion of the posterior shoulder by lifting the head toward the symphysis, at the same time making slight traction.

When the child is born have the nurse make gentle pressure over fundus of the uterus. If the child does not cry out, clear the mucus from throat and mouth with the finger, place the child in a basin of hot water, and dash cold water on the chest until breathing is established. A good plan is to introduce finger into its rectum; there are numerous other plans, but I believe these to be the best. Always lay the child on the right side, as this position favors the closure of the foramen ovale. After respiration is well established tie the cord, waiting a sufficient time for the blood to flow from the placenta before so doing. The first cord should be tied about three fingers' breadth from the umbilicus, the second about two inches from the first, nearer to the placenta.

There are three things to be considered in the third stage of labor: First, to assist in the delivery of the placenta; second, to keep up uterine contraction; and third, to prevent hemorrhage. I usually wait fifteen or twenty minutes after the delivery of the child before assisting in the delivery of the after-birth. I always use Crede's method in the expression of the placenta. After the placenta has been expelled into the vagina, traction may be made upon the cord, and extraction slowly accomplished, at the same time keeping up pressure upon the fundus of the uterus. If there is any indication or tendency to hemorrhage at this time, I administer a half-dram dose of ergot, otherwise I do not give any; but I make gentle pressure and some friction over the uterus for some thirty minutes after the escape of the placenta to keep up firm contraction.

To remove the vernix caseosa from the child I use warm lard or vaseline. After bathing the child it should be dried and dusted all over with borated talcum powder; after dusting the cord well place it on the left side, wrapped in a piece of soft cloth or cotton, then apply the belly-band, not too tightly, as the lungs will expand. After delivery the external organs of the mother should be cleansed thoroughly with an antiseptic solution, and repeated after this as often as necessary to keep up cleanliness. All soiled linen should be removed from bed and mother, and changed as often as necessary. I generally use a binder for the purpose of keeping up pressure over the uterus, and preserving the original figure of the patient.

In normal deliveries it is not only useless, but I consider it bad practice, to use vaginal injections. It is best to have your patient lie on her back during the first few days of her puerperium, and to remain constantly in bed for at least ten days. The physician must give specific directions in regard to rest and quiet, permitting only those necessary to wait on the patient to remain in the lying-in room for the first few days. If the after-pains are too severe, give ergot to produce free contraction of the uterus, and add opium to diminish the pain of contraction, as they are usually caused from a relaxed womb and accumulated blood-clots.

In extreme cases it may be necessary to give a hypodermic of morphine. The diet is matter of no small importance during the puerperal state. I prefer a light diet, chiefly of milk for the first few days, gradually increasing it until on the sixth or seventh day, when the patient may take any food suitable for a healthy person in bed. In this way you will avoid many of the inflammatory conditions about the genitals and breasts.

The physician is often annoyed by having to use a catheter after confinement to relieve the over-distended partially paralyzed bladder, which may be avoided if you will caution the nurse and patient to use the bedpan on the slightest inclination to urinate. On the second night after delivery give a purgative, and repeat on the following morning if necessary. After the mother has had some rest the baby should be put to the breast—it stimulates uterine contractions if nothing more. If the breasts become engorged, accompanied by symptoms of heat, pain, and fullness, give a saline p urge; put baby to breasts every two hours. If the secretions are excessive use breast pump, and have the nurse rub and massage the breasts toward nipple, using vaseline. You may apply cloths wet in lead-water and laudanum.

It is best for the obstetrician to remain for at least an hour after delivery, and in country practice, where we can not revisit the patient as often as our city cousins would under similar circumstances, be sure and leave specific directions for them to call you should any untoward symptoms occur—*American Practitioner and News.*

---

### USEFUL THERAPEUTIC MEASURES IN PHTHISIS.

BY M. SHELLENBERGER, M.D., PHILADELPHIA.

The sympathy between the lungs and stomach has been ascribed to a reflex nervous influence by which an undue secretion of gastric juice is excited, and the functional disorders of digestion which depend upon this may, in their turn, lead to catarrhal and inflammatory states of the mucous membrane. In the later stages of the disease the obstruction to the flow of blood through the lungs will keep the stomach in a constant state of passive congestion. A small exciting cause will then set up inflammatory action and symptoms of greater severity. A further question enters into the subject of the connection of dyspepsia with phthisis. The want of assimilation of fat has been adduced by some as one of the causes of the development of tubercle. Bennet, the originator of these views, lays stress on the fact that in phthisis "an excess of acidity exists [in the alimentary canal," and that, hence, by the neutralization of the pancreatic juice, fats and carbohydrate food are imperfectly prepared for secondary digestion and assimilation.

A dyspeptic origin is therefore given for all cases of phthisis, or, if that be not so, there is a fallacy in the argument which gives a primary place to imperfect fatty metabolism in the causation of phthisis, and yet attributes it to a condition of digestion itself dependent on phthisis. Fox regards the connection between phthisis and the perverted metabolism of fat as an indirect one in the incipient stages of the disease, and others regard the distaste for fat, which occurs so frequently in phthisis, as purely secondary to digestive disorders. Vomiting might be also declared to be the chief characteristic of phthisical dyspepsia. Habershon divides it as follows :

I. Peripheral or sympathetic vomiting.

a. Irritation of the peripheral terminations of the pulmonary branches of the vagus.

b. Irritation of the pharyngeal and laryngeal mucous membrane, including the epiglottis.

c. Interference with the trunk of the vagus by enlarged bronchial glands (adenopathic tracheo-bronchique).

II. Vomiting due to the violence of cough (so-called mechanical vomiting).

III. Gastric vomiting proper. (Irritation of gastric branches of the vagus.)

IV. Central irritation. (Tubercular meningitis.)

In the stomach itself there are three gastric affections due to phthisis :

1. Acute and chronic catarrh of the stomach.

2. Atrophy of the coats of the stomach.

3. Ulceration of the stomach, non-tubercular or follicular, and rarely tubercular.

In the treatment, therefore, it is a wise precaution to examine the lungs in cases of intractable dyspepsia. In cases of dyspepsia, it is necessary to insist upon the great importance of pure and bracing air, and of residence on dry soil, to supplement the medicinal treatment. Nowadays, with our numerous convalescent homes, this is not impossible even in dispensary practice.

Bland and an easily digestible food should be ordered. Nothing irritating to the mucous membrane of the alimentary tract should be allowed, either in the shape of food or drink—such, for instance, as condiments, vinegar, acid drinks, or alcohol in concentrated form. Should the vomiting become frequent or severe, it is hopeless to attempt to treat the patient unless at rest in a recumbent position. In such cases, also, liquid nourishment is advisable. This is obvious enough when the symptom is the result of gastritis, but it is equally applicable in the distressing vomiting that results from the cough occasionally.

Sympathetic vomiting, due to irritation of the pulmonary branches of the vagus, is a most intractable form, but it may subside as soon as the patient is kept in bed and put on liquid diet. Medicinal treatment alone without these precautions will invariably fail. The object should be to allay any irritability of the stomach that may be present, and bismuth in the form of carbonate or subnitrate, with bicarbonate of sodium and some sedative, such as opium or morphin, hydrocyanic acid, or bromid of potassium. Soda water is a valuable sedative, and is suitable either alone or with milk. It must be remembered that the tendency to vomiting, from this cause, is to cease after a time as the disease in the lungs progresses.

In the throat affection, soothing and slightly astringent gargles will do much to lessen the irritability, and this applies also to all forms of pharyngeal irritation, from simple catarrh to the more painful forms of ulceration of the soft palate or pharynx. Great advantage ensues from the use of a sedative gargle of bromid of potassium and opium in combination. To these add a mild astringent such as borax. Some cases of longer duration are benefited by more powerful astringents, such as alum or tannin, but these should be combined with opium or some topical anodyne. For ulceration of the fauces a powder of iodoform, borax and morphin, dusted or blown upon the ulcerated surface once or twice daily will frequently give relief. When the throat is not specially sensitive the difficulty of expelling the sputum often gives rise to exhausting efforts of coughing. Expectorant remedies combined with iodid of potassium (three to five grains are usually sufficient), or alkalies, will do much to aid in the expulsion of secretion. Opiates may be given in small doses when the amount of secretion is small and there is an undue irritability of the smaller bronchial tubes. If, as in some cases, there is any degree of spasm of the small bronchi, iodid of potassium and carbonate of ammonium in small doses are excellent. Ipecacuanha should not be present where the cardiac action is feeble. If all these internal remedies are sometimes unsuccessful, then try inhalation. For moist inhaling a sedative alkaline inhalation, such as the vapor coninæ of the British Pharmacopœia, or a mixture of the succus conii with ammonia (ten drops of the liquor ammoniæ to the pint of hot water) is good. For the cough, violent at night or in the early morning, dry inhalations are of more service from their greater convenience. Drops of the liquid remedy are placed upon the sponge or cotton wool in an oro-nasal respirator. Menthol or thymol dissolved in rectified spirit or spirit of chloroform may be used alone or in combination with compound tincture of benzoin and eucalyptus oil. A prescription in use in the Royal Chest Hospital is as follows:

| | |
|---|---|
| Ol. eucalypti | dr. 2 |
| Tinct. benzoin comp | dr. 3 |
| Thymol (or methol) | dr. 1 |
| Spirit chloroform | oz. 1 |

Ten drops at a time, on cotton wool in an oro-nasal respirator, are to be inhaled. Oil of peppermint alone is equally effective in lessening irritability, but is disagreeable to some on account of its smell. The most obstinate cases to deal with are those in which there is excessive irritability of stomach. Vomiting follows shortly after a meal, and is often preceded by a sense of suffocation, without pain and with or without violent cough. (Sometimes the cough is very slight.) A drug which Paget first suggested, and which Habershon frequently used with success, is liquor potassæ (five to ten minims for a dose), with sometimes a few minims of laudanum added. The success of this remedy is probably due to the fact that it counteracts the excessive acidity of the stomach which is so frequently present, which is sufficient to keep up a certain irritability of the gastric mucous membrane. It also has the property of alkalies of rendering more fluid the secretions.

Codrescv for uncontrollable vomiting injects hypodermatically one sixth to one fourth of a grain of morphin in the epigastric region, the patient being kept in bed or in the recumbent position.

Habershon has used this remedy with considerable success. In the case of C. B., a man aged about thirty-five years, food was rejected invariably from a few minutes to half an hour after it was taken. There was no evidence of gastric catarrh. The tongue was thinly furred, but not red or irritable in appearance. The throat was sensitive, but not inflamed. The pulmonary disease was confined to the left lung. No remedies appeared to have the smallest effect in reducing the vomiting, and the man was becoming rapidly emaciated from his inability to take food. He injected one fourth of a grain of morphin in the epigastric region, with the result that for the whole of the following week there was no return of vomiting. At the beginning of the second week he injected a smaller dose, one sixth of a grain. The patient vomited two or three times in the course of the following few days. In all cases of vomiting with the cough, the patient should be recommended not to exert himself after a meal, but to lie down for half an hour or an hour, while, as said, extreme cases should be confined to bed.

The nutrition of the patient is all important; yet drugs which improve nutrition are unfortunately not many. Among them arsenic is undoubtedly foremost, then comes strychnin—not in large amounts. These drugs have the constitutional effect of improving the blood and stimulating the circulation, respiration, and organic functions, yet the effect upon digestion should be our guide. Hence, arsenic and strychnin are doubly useful, both to improve digestion and to stimulate the blood and the nervous system. A happy combination is strychnin arsenate, combining small doses of arsenic acid with small doses of strychnin. It may be given in doses of gr. 1-128 to gr. 1-64. In the smaller dose, give in the average case six doses daily. The frequent administration of the smaller doses is the better if possible. Arsenic in Donovan's or Fowler's solution can also be given, or in the shape of arsenic iodid or sodium arsenate. In gastric irritability one-half pint to one pint of hot water about an hour before each meal, or the principal meal, is excellent. This cleanses the digestive tract and gently stimulates the mucous membranes. For marked gastro-intestinal disorder the special indications in each individual case must be treated. Lavage with alkaline solutions is useful in many cases.

Turpentine is important in intestinal catarrh in the form of oil of turpentine, terebene, or terpin hydrate. Ammonium chlorid can be used to alleviate gastro-intestinal symptoms, being often more useful than turpentine should gastric symptoms be prominent. Sodium phosphate is useful where a tendency to duodenal or hepatic catarrh exists, given in half-dram to dram doses, in hot water, half an hour before meals. Remember it has a laxative and occasionally a purgative action, hence the dose must be watched. For the fermentation in the alimentary canal, hydrogen dioxid water (three per cent.) in doses of two fluidrams, well diluted, before meals is excellent. Impairment of circulation producing indigestion requires digitalis, spartein sulphate, and similar remedies. The hypophosphites should occupy a prominent place combined with iron where there is diminution of hemoglobin or of red blood-cells.

Solis-Cohen suggests the following combination:

| | |
|---|---|
| Tincture ferric chlorid | dr. 2 |
| Acid phosphoric, dil | dr. 3 |
| Syrup hypophosphites (Churchill) q. s. ad | oz. 3 |

The dose being a dessertspoonful in water after meals.

The status of cod-liver oil is that of a food without other therapeutic value. It supplies fat when it is greatly needed, but if the patient is unable to digest fatty food, compound spirit of ether, one or two fluidrams before or just after meals, or of some preparation of the pancreas or of bile, is excellent.

To fight the toxemia of phthisis two remedies are especially valuable—creosote and iodin. Iodin can be given in various ways. Cohen, who has done much work in this line, prefers iodoform and ethyl iodid. Iodoform has a corrective influence over the processes of tubercle formation and when a patient can take it it is useful if pushed to the physiological limit. In capsules or in pill form with glucose, Cohen recommends the following:

| | |
|---|---|
| Reduced iron | gr. 1 |
| Iodoform | gr. 1—4 |
| Arsenic iodid | gr. 1-24—1-12 |
| Balsam Peru | gr. 2½ |

Dose one capsule after food thrice daily.

Unfortunately many patients cannot take this at all, but it is true that a patient taking iodoform pushed to the point of tolerance will recover considerable strength and weight.

Iodin as calcium iodid, as arsenic iodid or strontium iodid, as compound tincture of iodin, as syrup of hydriodic acid or by inhalations as ethyl iodid, can be tried. As a rule iodin in the early stages of chronic forms of pulmonary tuberculosis, especially in scrofulous subjects, is excellent, and even in advanced cases when there is but slight tendency to cavity formation. Creosote is the other drug for toxemia pushed nearly to the point of tolerance. When given in small doses, such as one half minim three times a day, before meals, it is a corrective to the stomach disorders; but against tubercular toxemia it should be given in comparatively large doses in milk. Success with creosote may depend upon the brand of creosote; much of the stuff that is sold for creosote is impure. Hence be sure you get the genuine article. If creosote cannot be given in milk, give it in capsules. The combination with morrhuol put up in capsules by Chapoteaut, of Paris, is most excellent. The guaiacol salts are excellent but expensive where creosote is not borne.

To affect the respiratory tract by inhalation of iodin, ethyl iodid or hydriodic ether can be given in this way: Take a wide-mouthed four-ounce phial, put in a tablespoonful of water, and drop into that five drops of ethyl iodid, which will sink to the bottom; then, as the drug is volatile, the warmth of the patient's hand as he grasps the phial will cause the vapor of the ether to rise through the water, diffusing through the air above it, and the patient can place the bottle to his mouth and inhale. This method may be used when you wish the patient to have a measured dose of the iodin: Let the patient take in his hand an ounce phial containing originally one half ounce of ethyl iodid, and inhale by simply holding the unstopped bottle to his mouth or nostril. It should be an amber-colored bottle, and, when not in use, kept cool and away from the light, to prevent decomposition of the drug. Let the patient inhale the medicament through the nose and mouth from one minute to five minutes, guaging the time by his susceptibility. Some persons become vertiginous in a short time, some in two or three minutes, and others not until after quite a long time. By this method there is systemic absorption of iodin, and the drug likewise exercises a useful influence upon local inflammations and ulcerations in the respiratory tract, especially in the larynx, both when inhaled and later when eliminated. Part of the usefulness of the terebinthinates, of creosote and of iodin preparations, when given by the mouth, skin, or rectum, is likewise due to local effect during elimination by the respiratory mucous membrane. Some physicians oppose inhalations in pulmonary tuberculosis because the method does not bring the drugs in contact with the bacilli in the lungs ; but these drugs are not employed with any such purpose. They certainly do not act by killing tubercle bacilli, either in elimination or in absorption, but they nevertheless do good. Don't get the idea that the purpose of inhalations or of antitoxemic medication is bactericide, for it is not. Action upon the cells and fluids of the body is what is aimed at, and while we cannot at present explain that action, its effects can be clinically recognized. A convenient method of inhalation is by means of the little perforated zinc respirator devised by Dr. Burney Yeo, of London, or a similar applicator made of perforated celluloid. Creosote, calyptol, thymol, terebene, menthol and chloroform are useful, by inhalation, in relieving cough, promoting the healing of laryngeal and bronchial catarrh, and disinfecting the contents of bronchiectases. For use on the sponge of the Yeo respirator, a mixture of equal parts of creosote, terebene, ethyl iodid and spirit of chloroform can be tried. Twenty drops is about the quantity used at one time, and it is renewed about twice daily. In cases of laryngeal ulceration, menthol (20 per cent.) in olive oil has been useful topically.

In all cases tact and perseverance are necessary, but the physician will feel repaid for his work in these discouraging cases.—*Medical Standard.*

---

### ON SYPHILIS.

BY JONATHAN HUTCHISON, LL.D., F.R.C.S., F.R.S.

It is now well recognized that syphilis stands side by side with the exanthemata, that it is a febrile disease, having quite definite stages, and that it must have necessarily from that fact a particular virus which breeds in the blood and produces the various phenomena associated with the disease. The stages have been classified, as all know, into primary, secondary and tertiary. That classification will never be set aside, although it must be fully recognized that it is not one which can be applied with very great strictness. We know what is meant by the primary stage of syphilis, namely, the stage of inoculation or introduction of the virus, characterized usually by the formation of what is known as a primary sore, a chancre. The secondary stage is the febrile stage, during which an eruption comes out on the skin of the patient and on the mucous membranes, and during which there is a certain amount of febrile reaction. So the primary or first stage is a local one, and the second is a blood stage, in which the virus is in every portion of the blood. The secondary eruption will be accurately symmetrical, proving clearly that it is a blood disease. In the third stage, to which the name tertiary is given, the symptoms are no longer symmetrical, they no longer imply blood disease, and the patient is no longer capable of communicating the virus to any one else. The symptoms of the tertiary stage concern the solid tissues and not the blood.

We will now consider the peculiarities which attend the inoculation, or, in other words, the primary sore. We all know that what are called chancres differ very much in their features. In book descriptions of chancres it is sometimes implied that the primary sore of syphilis is always indurated and sclerosed. There are very many exceptions to this. Syphilis does tend to produce very remarkable sclerosis in some cases, but it does not do it in all, nor does it do it in all parts. For practical purposes the most definite examples of sclerosis in connection with a primary sore may be said to be met with only on the genitals. It is said that on the female genitals you scarely ever meet with indurated sores. That is a mistake. They are frequently overlooked and they are seldom so characteristic as those met with on the genitals of the male. On the genitals of the male we often meet with a form of induration, especially just above the corona, as hard as cartilage, which is quite specific, and which, to those who have become acquainted with it, is pathognomonic. Fifty years or so ago, the distinction between the hard and the soft chancre was strongly drawn—that is to say, the distinction between what I prefer to call the infective and the non-infective chancre. We do not nowadays hear anybody talking about dualism in syphilis; we recognize that there are sores which are non-infective as well as those which are infective, but that there are two kinds of syphilis and that the word dualism is in any way applicable no one nowadays dreams of. In investigating syphilis we are not dealing with the introduction of a pure virus; there is no such thing as a pure culture in the case of syphilis. It is introduced in a mixed state in the great majority of instances, and it is owing to that mixture of pus and other secretions, and to the accidental way in which syphilis is conveyed that we owe the multiplicity of appearances in the primary sores. The incubation stage, prior to the appearance of any sore, before the place which has been inoculated will inflame in any degree whatever, will be at least three weeks, probably a month, and possibly five weeks. The sore on its first appearance will be simply a little papule or red patch or spot which may be observed for a few days. It will take a week before anything in the nature of specific induration, which you can estimate with the finger, will be produced. If no sore has appeared until the end of nearly four weeks, it is probable that it will indurate within a week of its beginning to such a degree that it may be recognized as an infective chancre. In some cases other sores due to the introduction of the products of inflammation, pus and other poisons are present, and the elements which the pus may contain may produce a sore which may take precedence of a true chancre. A patient may have a non-indurating chancre which was never at any stage "hard." The absence of induration is all that we should take cognizance of; "softness" is not in itself a positive feature, not anything pathognomonic, to which we can trust. For practical purposes I warn every one most strongly never to tell a patient who has got a sore which may possibly have been contracted by a venereal source that he has not got syphilis until a month has elapsed, because it is not till then that you can judge; the syphilis may be absolutely latent, or it may be entirely concealed by the presence of something else, such as an inflamed sore, until that period has elapsed. Hence my idea of the relationship between the nonindurated sore and indurated. The sores which are not indurated are the result of various forms of pus contagion, and they may very often carry with them the virus of syphilis.

The peculiarities presented by the sores called "soft" vary very much indeed. Multiplicity is one of the features in which the non-indurated sore differs from the indurated. The secretion of pus is another; but an indurated sore may also be multiple, and four or five up to fifteen indurated sores have been noticed together. On the other hand, non-indurating sores may be single. But in proportion to the shortness of the period intervening between the contagion and the appearance of the sore, so may you feel sure that the sore is not as yet syphilitic. This does not exclude syphilis afterwards, but it makes it pretty clear that that sore, as at present seen, is not due to the syphilitic virus, that virus producing nothing until at least three weeks have elapsed. In the true indurated Hunterian chancre it is usual for the glands in the groin to be enlarged and to become very hard. The expression "bullet bubo" is a very proper one. The chancre may be as hard as cartilage, and the swelling in the glands of the groin may be as hard as bullets, quite movable, that is, not becoming glued together. Here again the tendency of the syphilitic virus is to cause an adhesive inflammation with fibrinous exudation, a tendency to organization and not to suppuration, whereas in the other form there is a tendency to suppuration. But just as the sore itself is inflamed and ulcerated, so the bubo, which results from it, is inflamed and tends to suppurate. But none of these features of distinction must be pushed too far. The bubo of true syphilis may also suppurate, and this we witness not very infrequently. There is a chance also, of course, that the other form may not suppurate; indeed, it is only exceptional that it does so.

A useful point in diagnosis is to examine the glands. If you find hard glands in the groin the inference is there has been a chancre at the anus, on the perineum, or on the genitals, and if you find a hard bubo in the armpit, that the hand has been the seat of infection. Whilst I believe that there is such a thing as syphilis without any obvious chancre, I have no doubt there is in most of these cases some trivial sore, and it must in some be trivial to a minute degree, because observant patients and surgeons often fail to identify it. In the cases of surgeons who get syphilis after having their hands exposed in midwifery practice, and who yet never recognized a chancre, I suppose the virus has probably been lodged by the side of the nail. Surgeons have become the subjects of syphilis from rather deep pricks with needles which were poisoned. In one case a surgeon had pricked himself in the thumb with a needle when operating on a syphilitic patient. The site of the prick was a little tender for some time, and then there was just the slightest brown discoloration around it, and that was all he had in the way of chancre. In a great many cases the sore is not a characteristic one; we are far too much in the habit of insisting that the primary sore of syphilis should always be a well characterized, indurated chancre. If we can have syphilis without a chancre in the case of pricks, it is possible it may follow sores on the genitals which never indurate. In the diagnosis of primary syphilis, we, of course, pay great attention to induration, for when it is present it is a symptom which is beyond appeal. If there is no induration whatever in the glands, that is another reason for doubting specific inoculation, but the omission of induration of glands is not infrequent in syphilis, and we must not, therefore, trust too much to it.

Any one who has any doubt as to the influence of mercury upon syphilis, or upon the sclerosis produced by it, may easily convince himself. Give a patient with an indurated sore mercury and it softens in a few days, then melts away. Stop the mercury and it indurates again, resume the mercury and it again softens. As to the question whether the chancre will disappear without treatment, I believe that a primary chancre will usually disappear spontaneously in a month or six weeks, but it is difficult in the present day to get facts which help in this direction, because everybody gives mercury as soon as the disease is recognized. The induration will certainly go away, except in the rarest cases, after a period of induration of a few months. The secondary stage of syphilis will often begin while the primary one is still existent. It is a rule if the patient be not treated, for the symptoms of blood-poisoning to begin to manifest themselves long before the primary induration has disappeared. The peculiar feature of indurated chancres is recrudescence. By recrudescence of the chancre I mean its spontaneous reappearance after a considerable period in the precise locality in which the first appeared without any fresh inoculation whatever. The induration induced is exactly like that of the original one, so that any one who is not familiar with the fact that a recrudescence is possible would certainly be inclined to doubt his patient's statement, and to believe that he had contracted a fresh sore. There are two or three other peculiarities which the primary sore may assume; one is that it may cause not a hard ulcer, but a fungating growth. This is important, because it has been this kind of sore which has been claimed as peculiar in a case of yaws. Whilst a primary chancre may sometimes never ulcerate at all, it is sometimes nothing but an ulceration. Primary chancres have been cut out in mistake for tumors because they were not ulcerating, the skin around remaining perfectly sound and soft.

It has been customary to say that the soft chancre never occurs anywhere but on the genitals, but as a matter of fact almost all sores which occur on the tongue, lips, skin or other parts are infective. These are not all indurated. I have seen on the fingers definite induration, but in the majority of cases you must not expect to recognize specific induration in an erratic chancre.

As regards the secondary symptoms and the period of their appearance in round figures, we will say that two months must elapse before the secondary symptoms begin. The patient at that time will become a little feverish and will probably have an eruption on the skin, and that eruption will perhaps be in the first instance roseolous, an eruption which very probably may be overlooked by the patient. That eruption is quite transitory and is usually followed in the course of two or three weeks by a papular or mixed one.

With regard to treatment, I entertain no doubt that mercury may be regarded as a specific against the syphilitic virus. It removes the indurated chancre and prevents the secondary symptoms. Of the latter fact I make no question whatever. The symptoms which it least constantly prevents is the sore throat; indeed, mercury often causes sore throat, but there will be no skin phenomena whatever. The inconveniences attending the use of mercury are the salivation and diarrhea. If a patient is dieted while he is taking the mercury he will not have diarrhea. Every kind of fruit and all green vegetables should be forbidden, and the patient should live simply upon beef, mutton, fish, and potatoes and bread, and an opium pill can be given if necessary to prevent diarrhea.—*Medical and Circular, January 7, 1903.*

---

### THE TREATMENT OF INOPERABLE CANCER OF THE UTERUS—A CRITICAL REVIEW OF RECENT LITERATURE.

BY GEORGE GELLHORN, M.D., St. Louis, Mo.

Inasmuch as the great majority of cases, when seen for the first time by the gynecologist, already belong in the category of the inoperable, palliative treatment of uterine cancer is almost as important as radical procedures. In such a case of carcinoma where radical surgical treatment is impossible, it must be our aim to adopt that line of therapy which offers the best means of making the patient's life bearable so long as it lasts. According to the site, the form and the extension of the disease, our choice will be influenced, yet, there is always a triad of symptoms which requires our interference—namely, hemorrhage, discharge and pain.

Cooper,[1] when speaking of the present treatment of inoperable cancer in general, surveys the relative merits of the many remedies that have been recommended. Neither subcutaneous injections of anticancerous serum or Coley's fluid (a mixture of the toxins of streptococcus erysipelatis and bacillus prodigiosus), nor parenchymatous injections of various irritating substances, such as alcohol, acetic acid, corrosive sublimate, methyl violet, venom of the cobra di capello, oil of turpentine, arsenious acid, etc., have been of any beneficial influence to the patients. Thyroid feeding, administration of drugs, such as chelidonium majus, and electricity, have proved equally useless. Cooper's skeptic view on the value of calcium carbid is supported by Chase,[2] who found that calcium carbid does not reduce odor nor hemorrhage nor give more comfort to the patient than other rational lines of treatment.

The effect of methylene blue on uterine cancer has been studied, among others, by Cucca and Ungaro,[3] who claim that this remedy not only diminishes hemorrhages and discharge but also relieves pain, so much so that the patients can do without morphin. The extension of the carcinoma is markedly delayed so that the life of the patient is considerably lengthened. George[4] confirms that, as a result of injections of methylene blue, a marked diminution in the size of the tumor was observed with some relief from pressure symptoms, "but there was an excessive stimulation to the growth of tumor-cells in the neighborhood."

It is only the X-rays and the Finsen light that, in Cooper's opinion, offer a good hope of improvement "in cases of inoperable rodent ulcer and in the superficial malignant ulceration on other parts." The literature on the effects of the X-rays and the Finsen light on uterine cancer is exceedingly meager contrasted with the overflow of reports on carcinoma in other regions of the body. Among the most recent papers on this subject, an article by Hopkins[5] may be mentioned. This article, however, though it deals in enthusiastic terms with the excellent results obtained, is not convincing, as it does not give any exact data and refers only to successful cases. It may not be altogether denied that in some few cases the application of X-rays has been followed by fair results. Grubbe[6] reported a case of recurrent inoperable cancer which, by this method, became "symptomatically cured." Coley[7] treated four cases of inoperable cancer. In one of these cases, a carcinoma of the cervix, the growth "apparently" disappeared; in the remaining cases there was little or no effect noticeable. My own experience is limited to a single case which did not show the slightest improvement after a thorough X-ray treatment of six weeks. "There is little ground," says Pusey,[8] "for hoping that the X-rays, as they must be applied at present, have more than a slight effect on malignant growths in the cavities of the body. There is some reason to believe that the use of the X-rays in such cases has an effect in relieving pain. And as X-ray exposure may be given these patients without disturbing them or interfering with their comfort, there seems no reason why they should not have the benefit of the remotest chance of relief."

But few words need be said about the cancroin of Adamkiewicz.

This author had, in several articles, claimed that a chemical fluid invented and prepared by him and termed "cancroin," could, when injected under the skin, definitely cure any form of cancer. His latest report,[9] however, was soon followed by a number of protests from other observers—Nothnagel, v. Eiselsberg,[10] Jacoby,[11] Pulawski.[12] So far as the value of cancroin for uterine cancer, in particular, is concerned, Poten and Schultz-Schultzenstein,[13] established its uselessness.

The recent development of radical abdominal hysterectomy for cancer led also to a palliative operation in cases which were beyond the limits of radical surgical aid. This operation, first devised by Pryor,[14] was revived by Kroenig.[15] The method consists of the bilateral ligation of both the hypogastric and ovarian arteries and the arteries of the round ligaments in order to check hemorrhage and to stop the foul discharge. This procedure was recommended when, during the laparotomy, after the incision had been made, the carcinoma was found to be too far advanced for any radical operation. In these cases the abdominal cavity should not be closed without effecting the ligation of the main vessels which supply the uterus. Furthermore, the indication for this "slight" operation which may also be performed through two lateral incisions and without opening the peritoneal cavity, was extended to those cases in which impossibility of a radical operation was evident beforehand. So far, this operation has been performed twelve times by Pryor,[14] Kroenig,[15] Iwanow,[16] Biermer[17] and Lindenthal.[18] The immediate result in all of these twelve cases was encouraging in so far as the hemorrhage ceased at once; the discharge, too, was considerably diminished, especially when the ligation of the arteries was combined with an excochleation and cauterization of the carcinomatous tissues. In some of these cases of Iwanow and Lindenthal the pain was relieved, and the general somatic condition of the patient was improved when the pre-operative cachexia had not been too pronounced. On the other hand, the ligation of all the vessels supplying the pelvic cavity bears the danger of trophoneurotic disturbances, as shown in Biermer's case, in which locomotion was greatly impaired. Regarding the late result, the further extension of the new-growth cannot definitely be prevented, and the sloughing and hemorrhages return so soon as the collateral circle is established. The duration of this free interval varies, but so much as fifteen months may elapse before the former symptoms recur (Iwanow). The patients seem to bear the operation rather well, and as temporary relief is beyond question, the operation is indicated in some few cases; provided the vagina is not too far involved, the cachexia is not too pronounced, and the main symptom in the individual case is hemorrhage, which by other means could not be checked.

In order to temporarily do away with the sloughing and distressing odor of ulcerating uterine cancers, two operative methods have been devised, which, however, have not found many followers. Gottschalk,[19] as well as Kuestner,[20] perform artificial occlusion of the vagina (colpocleisis), the latter in addition producing a recto-vaginal fistula above the occlusion, thus allowing the discharge and blood from the uterus to flow into the ampulla of the rectum.

The foregoing survey of the recent literature on the treatment of inoperable cancer shows us that most of the remedies praised with so much enthusiasm during the last four or five years have not fulfilled their promise. X-ray treatment seems to offer some slight hope; as yet, the number of cases really benefited by this method is much too small to warrant any exaggerated expectations. Ligating the blood-vessels of the pelvic cavity presents some features that, so far, are rather encouraging; but this mode of treatment must be reserved for a minority of cases.

Thus, the endeavors and labors of the past lustrum have not been successful in changing materially or enlarging the older and time-proved mode of treatment of inoperable cancer of the uterus. The only surgical proceeding that has been resorted to during an extended period of time with any confidence and with a certain degree of success, is the use of the curette or sharp spoon, followed by the actual cautery or the application of a very strong solution of chlorid of zinc (Czerny,[21] and Sinclair[22]). The after-treatment aims to keep the wound cavity perfectly dry by application of either iodoform, tannic and boric acid, salicylic acid, aristol or a mixture of iodoform and charcoal. Especially the latter compound, which was first recommended by Torggler,[23] has, in my experience, proved very efficacious.

In cases in which excochleation cannot be performed on account of the danger of perforation into adjoining organs, or in which such a communication already exists, our treatment must needs be merely symptomatic. The application of such powders as have been enumerated above will for some time suffice to slightly diminish the unbearable odor of the discharge. But, as the vaginal walls, as a rule, do not stand very well dry treatment, vaginal douches will be resorted to sooner or later. Among the long list of drugs used in solution for douching, permanganate of potassium, thymol, alum and pyroligneous acid have been applied with advantage. Recently, peroxide of hydrogen in 3 per cent solution (A. Martin) or 12 per cent solution (Torggler), as well as formalin in various concentrations (Torggler,[23] Ranalletti[24]) is recommended. Both medicines have a deodorizing effect and, by hardening the surface of the necrotic masses in the vagina, lessen the discharge.

To relieve the pain, we may, at first, prescribe antipyrin or phenacetin, the latter of which is recommended by Lomer[25] as having a speciffic influence on cancer pain. Later morphin or heroin must be administered either in form of suppositories or hypodermatically. Considering the suffering of the unfortunate afflicted, one cannot but agree with the suggestion of Leighton[26] "to turn the patient into an unconscious opium-eater."

Copious water enemas relieve the frequent constipation better than laxatives. Diuretics will serve as good prophylactics against the diminution of urinary secretion. As to the rest, we ought to keep the patients out of bed as long as possible in order to avoid psychic depression and to prevent decubitus.

BIBLIOGRAPHY.

[1]Lancet, vol. 2, p. 964, 1901.
[2]Jour. A. M. A., June 22, 1901.
[3]Rassegna d'ost. e gin, Jan., 1901.
[4]Phil. Med. Jour., Jan. 11, 1902.
[5]Ibid., Feb. 21, 1903.
[6]Med. Rec., Nov. 1, 1902.

[7]Med. News, Jan. 31, 1903.

[8]Jour. A. M. A., April 12, 1902.

[9]Berliner Klin. Woch., p. 569, 1902.

[10]Ibid., p. 659.

[11]Deutsche Med. Woch., Jan. 2, 1902.

[12]Ibid., Nov. 6, 1902.

[13]Berliner Klin. Woch., pp. 660–661, 1902.

[14]Am. Jour. Obstet., p. 801, 1896 and p. 481, 1897.

[15]Centralblatt f. Gyn., p. 1073, 1902.

[16]Ibid., No. 4, 1903.

[17]Ibid., No. 7.

[18]Ibid., No. 10.

[19]Centralblatt f. Gyn., p. 79, 1899.

[20]Ibid., p. 360, 1900.

[21]Deutsche Aerztezeitung, Heft 10, 1900.

[22]Practitioner, July, 1902.

[23]Munch. Med. Woch., p. 1212, 1901.

[24]La Rif. Med., Nov., 1900.

[25]Ency. der Geb. und Gyn., p. 210, 1900.

[26]Brit. Med. Jour., March 16, 1901.

---

### INCREASE IN MEDICAL CORPS UNITED STATES NAVY.

The following items, as furnished by the surgeon-general of the navy, will be of interest to those who contemplate applying for position in the medical corps of the United States navy :

The last Congress provided for an increase of 150 members in this branch of service, twenty-five of whom are to be appointed each calendar year for six years. The number of vacancies occurring from retirements, resignations and casualties average about ten a year in addition to the twenty-five created by new legislation, thus making the total number of appointments necessary about thirty-five yearly. These appointments are open to any well qualified physician between the ages of twenty-one and thirty, who is a citizen of the United States. Examinations to determine the fitness of candidates are held by boards, which are in continuous session throughout the year at Washington, D. C., and Mare Island, California. Candidates should apply to the Secretary of the Navy for permission to be examined. No political or other influence is required, and the only testimonials needed are those bearing on moral standing and citizenship. The examinations are physical, professional and collateral.

The prospects of the medical officer of the navy, both for promotion and professional opportunity, are very bright, and the plan of enlargement of the naval establishment already adopted and authorized, as well as that in contemplation, gives assurance that this outlook will grow even more promising.

Appointments are first made to the grade of assistant surgeon (rank of lieutenant, junior grade), who, after three years' service as such, is eligible for promotion to the next higher grade—that of passed assistant surgeon—and these promotions continue at the end of five, ten and fifteen years in the service through the various other grades of surgeon, medical inspector and medical director. Pay varies from $1,402.50 on shore ($1,650 at sea) and $288 allowance per annum when quarters are not furnished by the government for the assistant surgeon, to $3,825 on shore ($4,500 at sea) and $720 allowance, etc., for the medical director (rank of captain). Eight cents a mile is the allowance when traveling under orders. The surgeon-general, ranking as a rear-admiral, receives $5,500 yearly, plus an allowance of $720.

The professional opportunities afforded the officers of the medical corps are very good, and are constantly improving. The first assignment to duty is usually to one of the fourteen naval hospitals, where he remains until the opening of the Naval Medical School in Washington, early in October. The Medical School is a post-graduate school, designed to fit the officer for the intelligent application of his professional knowledge to the requirements of the naval service and to give him a training in certain specialties peculiarly important to naval work. Five months are devoted to this school work, and after its completion the assistant surgeon is assigned to sea duty. He is provided with the latest and best instruments of precision and operation, and is given every encouragement to perfect himself in the practice of his profession. The most recently constructed battleships and large cruisers are equipped with hospital facilities equal to those found in most of our best organized small cities. Other duties to which naval medical officers are assigned are those pertaining to the needs of navy yards, naval stations, receiving ships and recruiting work. Opportunity frequently occurs also for attendance of medical officers upon the meetings of medical and other scientific societies both at home and abroad as the accredited representatives of the Navy Department and the government.—*Virginia Medical Semi-Monthly.*

---

### THE BEST METHOD OF ADMINISTERING QUININE.

McElroy, (Memphis Medical Monthly).

In speaking of quinine as an immunizing agent, Manson is quoted with approval: "The reputed prophylactic effect of quinine is but a phase of its therapeutic action; it is the application of the drug to the parasite, and not an immunizing of the body against the entrance of the parasite, one has to deal with; therefore one may confidently expect that if it will cure a malarial infection, it will prevent its development." Unquestionably if the quinine is present in the blood at the time the parasite is introduced it may prevent, but it is evident that it is much a matter of chance if the quinine is there at that time. It is, therefore, an uncertain prophylactic. It is rare that the spring tertian or the estivo-autumnal parasite as seen in our climate responds to five grains of quinine three times a day. The practice of giving small amounts of quinine with breakfast, dinner and supper, with a view to prophylaxis, is not effective and may be injurious, owing to the disturbance of digestion and the consequent anemia. It also interferes with the diagnosis of malaria, as after the administration of quinine there may be an inability to identify the parasites in the blood. If malaria has developed, the administration of fifteen grains of quinine once a week is effective in preventing the return of the paroxysms.

Quinine is a valuable therapeutic test. If administered to a malarious patient in whom the fever comes at fixed intervals, it usually causes an interruption of the cycle, and this is nearly as diagnostic of malaria as is the finding of the parasite. In the estivo-autumnal infections, five grains should be given every four hours. After the administration of thirty or forty grains the fever will remain down for several days. Quinine is of value in diagnosis in malarial districts, even where the microscope is available, as in some cases the malarial parasite is not found in the peripheral circulation, or it may be sought for patiently and not found, owing to the fact that very few parasites are present.

The sulphate of quinine is generally used because of its cheapness. To obtain prompt physiological results it should be given dissolved. The hydrochlorate is the preferable salt of quinine, having a larger percentage of quinine, and is less bulky. It is adapted for hypodermic use.

In regard to the subcutaneous use of quinine, the writer addressed a number of physicians living in the malarial districts of the South as to their experience with its hypodermic use. In the main the replies showed a confidence in the method. When the quinine is dissolved in normal salt solution and is not too concentrated, about ten grains to the half-ounce of solution, then the results are good. When the solution is concentrated or has an acid reaction, the results are *nil.* Twenty grains of bimuriate of quinine and urea and one pint of normal salt solution is an excellent mixture. One physician regards the hypodermic method as the only proper treatment of the pernicious forms of malaria.

From the replies received it appears that the intravenous injection of quinine is not often employed. The writer has used it three times with good effect. His experience is similar to that of Bacelli, who has successfully employed this method in the comatose forms of pernicious fever.

Its administration by the rectum has been so unsatisfactory that it has been discarded.—*Charlotte Medical Journal.*

---

### THE RADICAL CURE OF HYDROCELE.

Fangere (*Medical Press and Circular,* February 18, 1903) says that hydrocele, usually styled idiopathic, is not the least provoked by a lesion of the epididymis, as a careful examination of the organ would show; spermatic cysts, traumatism, rheumatism, syphilis, etc., only act through the influence of the gland. He would not go into the pathological anatomy or the symptoms of the affection, but he said a few words on the different varieties of hydrocele and the treatment. Besides the multilocular, diverticular, chylous, and milky varieties of hydrocele, there is the infantile or congenital hydrocele and hydrocele in woman. The infantile variety is generally attributed to a traumatism during delivery; it gets well spontaneously.

Congenital hydrocele; it should be considered as a vagino-peritoneal hydrocele, as in reality it is an accumulation of peritoneal serosity in the tunica vaginalis. It is called congenital malformation, and not because it is itself congenital, The lesion is due consequently to an arrest of development. In the normal condition the testicle descends into the scrotum, carrying with it a fold of the peritoneum. It becomes invaginated in the inguinal canal like the finger of a glove, and forms in this way the tunica vaginalis. That canal is temporary, becomes obliterated by degrees, and finally in its place is found a thin cord called the ligament of the vaginalis. At birth the canal exists still, establishing a communication between the vaginalis and the peritoneum, but the obliteration is effected rapidly in a few days. When the obliteration is not effected, congenital hydrocele is more frequent between the ages of three and ten, spontaneous reducibility in the horizontal position or provoked by pressure is the fundamental character of that kind of hydrocele, that benign affection which calls for treatment only because it favors the production of a hernia, is chronic in its evolution, and frequently disappears spontaneously, especially in the child, by obliteration of the canal.

Hydrocele in a woman consists in a serous cyst of the labium majus. As regards the treatment, numerous are the methods recommended; puncture followed by a modifying injection of tincture of iodine, iodoform and ether, nitrate of silver, or chloride of zinc. All these injections are more or less painful, and followed by considerable inflammation of the scrotum, the first and last named more particularly. The chloride of zinc solution is composed as follows:

| | |
|---|---|
| Chloride of zinc........................... | grs. xvj. |
| Water................................ | 3 drachms. |

After having removed one or two ounces of the liquid, from five to twenty drops of the solution should be injected by the ordinary hypodermic syringe. Inflammatory swelling is the result, but quickly subsides, and in two or three days the patient is cured. The method is much less painful than that of tincture of iodine.

The radical cure by operation is the best method of treatment, since antiseptics have so much facilitated the use of the knife. Several methods are employed—simple incision, partial resection of the tunica vaginalis, total resection of that membrane, and inversion as practiced by Doyen, and which he considers the best.

—*Charlotte Medical Journal.*

---

### ALCOHOL AS ALIMENT.

A recent essay on the part of the eminent French chemist Duclaux has had a somewhat disturbing effect on the minds of many to whom the alcohol problem is an important one. Basing his assertions on certain American research work, this eminent chemist has maintained that alcohol not only is not a poison, but that it is a food, which must be placed alongside sugar and starch, which substances it surpasses in alimentary value weight for weight. He continues his praise of alcohol by maintaining that this substance will shortly enter into all dietetic rations, and that we ought to make excuses for the obloquy we have heaped on the heads of those who in the past have dispensed this valuable food.

Such conclusions have a rapid circulation, and a leading chief of a factory has stated that his employees who had been convinced by the anti-alcoholic crusade of the harmfulness of this substance, now fortified by the dictates of science, are again frequenting the bars and saloons. The question is therefore one which has a profound sociological significance, and it is wise to consider on what grounds that which is condemned one day is recommended the next.

It would appear to be Atwater's work which has been the disturbing factor in these matters. The word "food" is the one which has passed with magical suggestiveness and upset conclusions previously arrived at in good faith. Atwater's experiments were concerned with the number of calories liberated by healthy men on a diet excluding alcohol. He found that alcohol furnished these calories, or as Duclaux states the fact: "In the alimentary ré-

gime of three heavy men it has been possible without inconvenience to replace butter, vegetables, and other analogous foods by alcohol in the form of wine or spirit."

Alcohol, weight for weight, can liberate more calories than other alimentary substances. This is the point which the public appreciates. In travel or under strain the substance which at least weight will furnish the greatest amount of energy is the one possessing obvious advantages. The calculation is simple: to liberate 100 calories it needs 24 grammes of sugar, 28 grammes of grain, 150 grammes of potatoes, or 150 grammes of milk. This can be done by 17 grammes of alcohol. This, however, is absolute alcohol, which would burn and injure the digestive organs, so the traveler would have to provide for the dilution of the substance he carries. Even then alcohol would not possess the merit of fat. Twelve grammes of butter would furnish the energy of 17 grammes of alcohol. Then, too, when we consider the bulk of alcohol, even as brandy, which is 55 per cent. absolute spirit, 24 grammes of sugar would be an equivalent in energy-producing results; so the traveler might consider this as the German army does, which simply tells the soldier to put a few lumps of sugar in his pocket, and this with excellent results.

The whole question is however something more than one of producing calories. Should we conclude that alcohol is a food, it is only so in this sense of producing calories, and it is costly and dangerous. If its use is suppressed we may have to replace it by other substances, and if we take at our repast a little more butter and sugar we can undoubtedly completely abandon the use of alcoholic beverages.—*Medical Age.*

---

### A WARNING TO COUNTER-PRESCRIBERS.

Counter-prescribing is a common vice but none the less objectionable. It is dishonest, in that neither the buyer nor the seller is competent to know the disease or the proper remedy; it is a violation of law, because the legislature has set its ban upon it; it is in its essence a fraud, because the remedies are advertised under fictitious names and with guarantees of efficacy and merit that are not founded on truth. There is scarcely a druggist whose counters are not covered with attractively labeled medicines for colds, headaches, kidney and other troubles which they do not hesitate to urge upon the buyer with all too persuasive eloquence. They are regardless alike of the harm that may follow, provided that they are not held financially responsible.

It is refreshing to see one of this class reaping the consequences of his deception. A recent case in New York, in which a druggist was obliged to pay heavy damages to one of his victims, was a vivid reminder of the responsibility which is assumed in counter-prescribing. The case does not differ from those which occur daily in every city. A man went into a drug store to purchase quinine for a cold, but was persuaded to take instead a proprietary remedy labelled "Dr. Goldsmith's Quick Headache Cure—Guaranteed Harmless." He took two tablets and two hours later took another according to directions. He was found unconscious in his office shortly afterward, dripping with perspiration, cyanotic, with respirations at the rate of four per minute and a pulse of fifty almost imperceptible. Stimulation and appropriate measures brought him back to health. He promptly sued the druggist for damages, alleging negligence and breach of contract, claiming that the guaranty of harmlessness of the medicine constituted a contract which was not fulfilled. It was elicited from the druggist on cross-examination that the tablets were "Migraine No. 2" purchased by wholesale and put up by him with fancy labels bearing the name of a fictitious Dr. Goldsmith. The clerks were paid a commission on all sales of this preparation and this probably acted as a stimulus to their deception of customers. A verdict for $3,281.00 was rendered against the druggist, the judge ruling only on the point of breach of contract.

This case is cited at length not only for the purpose of showing the methods which certain druggists use in deceiving the public as to the character of their so-called proprietary articles, but also to illustrate the dangers which they run of pecuniary damages in case of accident, as well as the actual wrong in indiscriminately placing at the disposal of the public drugs which may be dangerous. No one, other than a physician, familiar with the disease conditions present in his patient, should have the temerity to prescribe. The law specifically defines the prerequisites for the practice of medicine. Counter-prescribing is a violation of that law, and should be punished as such. The heavy damages awarded in this case were punitive damages, and should serve as a wholesome lesson to those reckless persons who are ambitious to increase their sales by fraud, deception and misrepresentation.—*St. Paul Med. Journal.*

---

### RAILWAY ACCIDENTS.

The Norfolk and Western Railway Company issued the following instructions to be observed in case of accidents:

"Employees will observe the following medical directions in cases of accidents:

"A. In accidents to persons, the ranking employee of the road present will take command and direct proceedings for the relief of the injured.

"B. When there is danger from fire, remove all persons promptly from the train, looking first to those who may be helpless from injury or jammed in the wreck.

"C. Take hold of the injured gently but firmly and without fear. Lay the injured one down on cushions, blankets, clothing or straw, where he will have perfect ventilation and not be in a draught or strong current of air. Loosen the clothes about the neck and body to permit easy breathing, and place the injured part in the position most comfortable to the sufferer. Do not permit strangers to approach and talk to or ask the injured one questions. Place him, if possible, in charge of one or two friends and keep him warm with proper covering.

"D. As soon as practicable, summon the nearest surgeon of the company and notify the superintendent by telegraph. State the number of persons injured and the nature and extent of the injuries, as clearly as time will allow, in order that the surgeon may come with what is needed.

"E. Bleeding.—If the bleeding is from the limbs, keep them bent and the bleeding points elevated as much as practicable.

"F. In case of broken bones, place the injured part in the most natural position, or, if this cannot be done, then in the position most comfortable to the patient. Having done this, seek to steady the limb either by splints of wood or by a pillow folded around the limb and tied in the desired position. In case of broken ribs, relief will be afforded by a wide bandage around the chest drawn as tightly as can be borne. When a broken bone is suspected do not move the limb about to find out if this is so.

"G. In cases of burns or scalds, cover the part with a paste made of baking-soda and water.

"H. When there is much weakness from injury, whisky may be given in small quantities, say from one to two tablespoonfuls, to be repeated at short intervals, if necessary. Large quantities must not be given, and no whisky must be given if the head is injured. In all cases of weakness from shock or loss of blood keep the patient warm.

"I. Cold water, ice, tea, coffee, milk or soup may be freely allowed to all injured ones who wish them.

"J. In moving an injured person, place a board, door shutter or mattress, with one end at the patient's head, and lift or slide him gently on it. If the patient can sit up, he may be carried in a chair or upon the locked hands of two persons, around whose neck, he throws his arms to steady himself.

"K. When forwarding a patient who has been seen by a surgeon, obtain from the surgeon a written statement as to his opinion of the nature and extent of the injuries, and attach this statement, along with the name of the injured one (if it can be obtained) securely to his clothing.

"L. When the injured person is able to be moved, take or send him to the nearest surgeon of the company in the direction in which the first train is moving. It can then be decided whether the patient will be treated there or taken to some other point.

"M. When the injured person is not able to be moved, place him in charge of the station agent, section master or some official of the road, and summon the surgeon of the company most easily obtained.

"N. In urgent cases, if no surgeon of the company can be promptly had, summon the nearest physician to take charge of the case until the company's surgeon arrives.

"O. In a general emergency, summon the surgeons of the company in both directions and wire the superintendent if more surgeons are needed."

It should be made an imperative rule in every factory that all injuries must be reported to the foreman at once, no matter how small or trifling. He or his assistant will apply the necessary first aid. If in any way doubtful as to recovery, the case should be referred by him to a competent surgeon, not the nearest, but the best.

Chewing tobacco, dirty cobwebs, soot, mud, turpentine, coal-oil, black oil, or any other oil, arnica, dirty rags, handkerchiefs in use, or rancid salves should never be used as a dressing for any wound.

Syncope or faint, fright and bleeding are the first difficulties to overcome in injuries. When your subject falls let him lie until he becomes conscious; then give him a drink of water, not whisky. Never apply ammonia or any strong stuff to his nose, nor sprinkle him with water; such treatment does no good. When the injured person is able to sit up wash his face and ears, and, when you observe him getting pale, make him stoop over low so his ears get red; he will then perspire and feel better and his faint goes away. Should it return, repeat your experiment. Encouragement and kindness is the most successful remedy for the frightened; especially children should be handled carefully. Never add to their misery with cruelty and unkind remarks.

Bleeding is the bogy man of surgery. Don't get scared by blood; when too severe, just hold the cut shut with your fingers and it cannot bleed.

Don't try to stop the bleeding by cutting off the circulation between the heart and injury. That is an old surgical superstition that generally causes more bleeding than if left alone, because it cuts off the veins. Tie the wound shut securely, as we will instruct you later in this series. A recent report of a first-aid surgeon shows that in over 36,000 injuries of all varieties, principally glass cuts, which bled severely, none have bled to death.

Pain is seldom very severe except in burns and smashed wounds that don't bleed. When finger- or toe-nails are smashed and have pounding pain and turn black, drill a hole into the nail with a sharp pocket-knife to let out the blood and pain readily ceases.

To correct deformity in injured parts, pull or squeeze them into shape as nearly natural as possible, applying some tension or force.

---

### METHODS WHICH RENDER SOME THERAPEUTIC AGENTS MORE PALATABLE.

A knowledge of the physiological and therapeutical action of a remedial agent is necessary in order to obtain good results; but there is one more thing needful, and that is to render it palatable so far as possible. The appearance and odor might also receive consideration; however, sometimes it is advisable to disguise the taste even at the expense of its attractive appearance. Of the two evils, choose the lesser one. If unpleasant medicine is made more inviting, it is an important factor in the management of the diseases of childhood and is also more acceptable to the adult.

To give castor oil or cod-liver oil in coffee or milk creates a prejudice against these beverages, and the taste of the oil is still prominent. Whisky and glycerin, given with each dose, may partly answer the purpose, or either one separately; however, some object to the whisky for moral reasons.

To give castor oil in one dose as a purgative, probably the "castor oil sandwich" is the best method. It is made as follows: In the bottom of a glass put a small quantity of glycerin, then the oil, lastly half an ounce of sherry wine, and take at one draught. This will also apply to the single dose of cod-liver oil.

In case either agent is dispensed in quantities, an emulsion in which the flavor of cinnamon or gaultheria predominates generally serves the purpose. Perhaps every one is familiar with the story of the young woman who asked a soda fountain clerk to prepare a dose of castor oil for her and also a glass of soda, and when she was informed later that she had drunk the oil with the soda, replied, "but the oil was for my sister." While the soda in this instance acted well, so also will it answer as a vehicle for Epsom salts, but the ordinary "soda pop" is better.

The attempts to disguise quinine have generally been only partially successful. Chocolate, yerba santa, and licorice in the form of a heavy syrup may be used, but I think the most preferable to be one grain of tannic acid to each three grains of quinine in a vehicle of syrup of tolu. The iodide and bromide of potassium and salicylic acid may be given in milk, which also prevents gastric irritation. However, some prefer in the case of iodide derivatives to use the iodated starch of the liquor iodi compositus. In case copaiba and turpentine are not used in gelatin capsule form, an emulsion flavored with gaultheria comes next in order.

For chloral hydrate I think peppermint water superior to cinnamon. Equal parts of peppermint water and simple syrup make the best solution for salicylate of sodium. Unless there is an objection to the intensely sweet taste, the syrup of glycyrrhiza answers best for sodium salicylate.

If the mouth is flushed quickly with a small quantity of whisky, the medicinal oils may be taken immediately afterward, and the disagreeable taste is not so perceptible.

A few grains of table salt taken upon the tongue will produce a copious flow of saliva, and then if swallowed with medicine which has an objectionable taste it may in a measure be disguised. Care should be taken, however, that no chemical incompatibility exists.

If lemon ice is held in the mouth for a moment only, then a teaspoonful of a preparation which would otherwise seem nauseous may be taken with very little unpleasant effect. In the last instance it is understood that both the medicine and the ice are swallowed at the same time.

Quite frequently medicine is taken into the mouth when the secretions are inactive, and therefore the membranes parched and dry, thus giving an opportunity for the bitter principles to remain in contact with the tongue and create an immediate unpleasant taste, but more especially an after-taste.

Sometimes simply a drink of water will obviate this condition, or perhaps a lump of ice held in the mouth, or water acidulated with dilute phosphoric acid, to be taken before the medicine. A

combination of syrup of red raspberry and glycerin makes an unusually palatable vehicle.

At a time when salicylate of sodium was unknown, it was very popular to combine sodium bicarbonate and salicylic acid, and the last mentioned menstruum is considered the best for this combination. In fact, I might include its use for the administration of sodium salicylate.—*S. E. Earp, in N. Y. Med. Jour., April 11, 1903.*

---

### RABIES, RAPID DIAGNOSIS OF.

Hydrophobia presents certain features which are of unusual interest and importance. Aside from the futile efforts to discover the causative agent, the pathology of the disease has been investigated by a number of observers with no better success. No appreciable lesions which could be considered at all specific have until late been brought to light, and the only pathological alterations found are confined to the nervous system.

This is not the least surprising, in view of the fact that hydrophobia is entirely a nervous affection, the rabic virus or toxin possessing a special affinity for the nervous tissue. In this respect it closely resembles tetanus, and it is quite possible that also in rabies the causative agent, whatever it is, invades the tissues locally and produces a powerful toxin which affects the nerve-centers.

However, the discovery of a specific lesion is more than a merely scientific problem. On it depends the diagnosis of this disease, and consequently the more rapid and satisfactory treatment. It is evident that the usual method of diagnosis of rabies by means of animal inoculations is entirely too slow a process to be depended on for practical purposes. As a rule, physicians do not wait with the Pasteur treatment until the diagnosis of rabies in the suspected dog is confirmed by animal inoculations, and the latter possess only a scientific value. This urgent necessity for a rapid diagnosis spurred various investigators to the effort of discovering specific lesions in the nervous system, where they would naturally be expected to be present.

Preceded by Benedict, Kolesnikoff, and others, Babès described an accumulation of leucocytes around nerve-cells which he called the "rabic tubercle" and which he considered pathognomonic of the disease. This claim, however, while confirmed by a number of observers, was contradicted by others, and even Babès himself has of late retracted his original views. The consensus of opinion is that Babès's "rabic tubercle" is secondary and inconstant in its appearance. Lately, Van Gehuchten and Nelis described certain pathological alterations, in the cells and capsule of the spinal ganglia which they consider specific. These changes consist principally in disintegration of the nerve-cells, chromatolysis, and proliferation of the endothelial lining of the capsule. The observations of these authors were fully confirmed by Hebrant, Nocard, Cuille and Vallée, Bailey, and especially by Ravenel and McCarthy. The latter observers have made a thorough study of these lesions, and so confident are they of their specific nature that they depend almost entirely on their presence for diagnostic purposes. Their position, however, is somewhat weakened by the fact that the same or closely similar lesions were discovered in other affections by Crocq, de Buck and de Moor, and Spiller. Furthermore, the diagnostic value of the lesions is also impaired by the fact that they may be, and usually are, absent in the earlier stages of the disease. Krizshanovsky (Archiv Biologitcheskich Nauk, vol. ix, No. 4, 1902) investigated the cardiac ganglia of rabbits, dogs, and men (two), dead of natural or experimental rabies. In no instance did he find the "rabic tubercle" of Babès, but the changes described by Van Gehuchten and Nelis he observed in all cases, the intensity of the pathological changes being in direct proportion to the chronicity of the disease. Proliferation of capsular endothelium and the invasion of the latter into the spaces formerly occupied by the nerve-cells he observed only in chronic cases. He concludes, however, that these lesions are not specific.

While there is still considerable uncertainty as to the nature of these lesions and their diagnostic value, the conclusion seems to be fully justifiable that, when other canine affections can be excluded by the aid of the history and a careful *post-mortem* examination, the presence of the ganglionic changes is fairly certain evidence of the existence of rabies.—*Editor Philadelphia Medical Journal, March 14, 1903.*

---

### THE FUNCTION OF THE DUCTLESS GLANDS.

A tremendous activity in collecting scientific data is manifest everywhere, and the practitioner despairingly throws up his hands when given access to this enormous aggregation of medical facts. We need some genius to formulate general laws, and to bring far-distant facts into a definite class.

One who has attempted this and boldly set forth certain general principles is Sajous. We take the liberty of analyzing a few of these theories.

"The thyroid gland, the anterior pituitary body, and the adrenals are functionally interdependent, and constitute a system, the 'adrenal system,' which has for its purpose to sustain physiological oxidation and the metabolic activity of tissues."

The author is entirely too radical in assuming that all metabolic processes consist in oxidation, and concluding from the clinical phenomena of myxedema and cretinism that this system presides over oxidation in general.

"All general symptoms witnessed in disorders in which the blood is involved by a poison of any kind are, in reality, manifestations of overactivity, insufficiency or inactivity of the adrenals."

This is another revolutionary statement, and displaces all the recent work on the effect of toxins on the blood and various organs. The function of oxidation is made more definite by the following aphorisms:

"When the venous bloods reaches the pulmonary alveoli, the marked affinity of the adrenal secretion in the plasma for oxygen causes it to absorb this gas from the alveolar-air. The carbon dioxid in the blood is thus forcibly replaced by oxygen and expelled with corresponding vigor. The red corpuscles, after this operation, bathe in an oxygen-laden medium, and then hemoglobin becomes converted into oxyhemoglobin."

"The physiological function of the internal secretion of the adrenals is loosely to combine with the atmospheric oxygen in the lungs and to endow the blood plasma with its oxidizing properties." The author ventures to call the oxidizing substance formed in the lungs "Adrenoxin."

The existence of a substance in the blood plasma which combines with oxygen has been suspected. In fact, Smith, on theoretical grounds, claimed that the lungs secrete an acid, pneumic acid, which unites with the alkali that is bound to the $CO_2$ in the blood and thus the carbon dioxid is displaced. Again, the oxidation of tissues to which the redblood-corpuscles can not go is explicable on the theory that the plasma is rich in oxygen. The adrenoxin may also take the place of Traube's "oxygen carrier" and thus form the intermediate body in oxidation. This is similar to the catalytic action well known to chemists.

But the evidence offered is too meager and while the existence of such a substance in the blood is rational, it is going beyond experimental physiology to state that it is the adrenal secretion.

REFERENCES.

Sajous.—Phil. Med. Jour., March 7, 1903.
Month.—Cycloped. Prac. Med., March, 1903.

—*Courier of Medicine.*

---

### Hemoptysis as an Initial Lesion in Tuberculosis.

F. Reiche (*Zeitschrift für Tuberculose*, Bd. 3) says it has long been known that consumptives with initial hemoptysis usually resort to a cure earlier and generally show a better prognosis. Reiche brings some statistical material to verify this statement. He saw for an insurance company up to the end of 1902 about 2,000 consumptives, of which 9.2 per cent. had an initial hemoptysis. He selected from these the most completely cured—*i. e.*, those who after twelve months evinced no sign of the disease—and found that the percentage of those having an early hemoptysis was greater amongst the cured than amongst the total number of cases. The condition is this: the hemoptysis is the signal for early treatment, thus favoring a good prognosis, though repeated hemorrhages are of themselves deleterious.—*Medical Age.*

